# A human tissue map of 5-hydroxymethylcytosines exhibits tissue specificity through gene and enhancer modulation

**DOI:** 10.1038/s41467-020-20001-w

**Published:** 2020-12-02

**Authors:** Xiao-Long Cui, Ji Nie, Jeremy Ku, Urszula Dougherty, Diana C. West-Szymanski, Francois Collin, Christopher K. Ellison, Laura Sieh, Yuhong Ning, Zifeng Deng, Carolyn W. T. Zhao, Anna Bergamaschi, Joel Pekow, Jiangbo Wei, Alana V. Beadell, Zhou Zhang, Geeta Sharma, Raman Talwar, Patrick Arensdorf, Jason Karpus, Ajay Goel, Marc Bissonnette, Wei Zhang, Samuel Levy, Chuan He

**Affiliations:** 1 Department of Chemistry, Department of Biochemistry and Molecular Biology, Institute for Biophysical Dynamics, University of Chicago, Chicago, IL USA; 2 Howard Hughes Medical Institute, University of Chicago, Chicago, IL USA; 3 Bluestar Genomics Inc., San Diego, CA USA; 4 Department of Medicine, University of Chicago, Chicago, IL USA; 5 Department of Preventive Medicine, Northwestern University Feinberg School of Medicine, Chicago, IL USA; 6 City of Hope Comprehensive Cancer Center, Duarte, CA USA

**Keywords:** DNA methylation, Epigenomics, DNA metabolism

## Abstract

DNA 5-hydroxymethylcytosine (5hmC) modification is known to be associated with gene transcription and frequently used as a mark to investigate dynamic DNA methylation conversion during mammalian development and in human diseases. However, the lack of genome-wide 5hmC profiles in different human tissue types impedes drawing generalized conclusions about how 5hmC is implicated in transcription activity and tissue specificity. To meet this need, we describe the development of a 5hmC tissue map by characterizing the genomic distributions of 5hmC in 19 human tissues derived from ten organ systems. Subsequent sequencing results enabled the identification of genome-wide 5hmC distributions that uniquely separates samples by tissue type. Further comparison of the 5hmC profiles with transcriptomes and histone modifications revealed that 5hmC is preferentially enriched on tissue-specific gene bodies and enhancers. Taken together, the results provide an extensive 5hmC map across diverse human tissue types that suggests a potential role of 5hmC in tissue-specific development; as well as a resource to facilitate future studies of DNA demethylation in pathogenesis and the development of 5hmC as biomarkers.

## Abstract (web summary)

DNA 5-hydroxymethylcytosine (5hmC) modification is associated with gene transcription and used as a mark of mammalian development. Here the authors report a comprehensive 5hmC tissue map and analysis of 5hmC genomic distributions in 19 human tissues derived from 10 organ systems, thus providing insights into the role of 5hmC in tissue-specific development.

## Introduction

Epigenetic modifications of cytosine residues on DNA play critical roles in development by modulating gene transcription. The most studied cytosine modification is 5-methylcytosine (5mC), which is associated with repression of gene expression. Specifically, the 5mC silences repetitive sequences, facilitates X-chromosome inactivation, and influences genomic imprinting in mammals^1^. The 5mC marks can be removed by either passive dilution through DNA replication or active demethylation by the ten–eleven translocation (TET) family of enzymes. In active demethylation, the TET enzymes depend on iron(II)/α-ketoglutarate to oxidize 5mC into successive intermediate states, including 5-hydroxymethylcytosine (5hmC), 5-formylcytosine (5fC), and 5-carboxylcytosine (5caC)^2,3^. Further excision by thymine DNA glycosylase (TDG) and the base excision repair (BER) pathway finally converts 5fC and 5caC into an unmodified cytosine^4^.

Over the past decade, studies have revealed that the oxidized forms of 5mC, particularly 5hmC, may have multiple independent functions beyond serving as an “intermediate” in the active demethylation process^5–9^. Unlike 5fC and 5caC, certain 5hmC modifications can be stable epigenetic marks during the cell cycle^10^. During early embryonic development, most 5hmC modifications are derived from de novo methylated 5mC instead of from the pool of existing 5mC, suggesting that the 5hmC may play a specific role during development^11^. Indeed, the high levels of 5hmC observed in embryonic stem cells and neuronal cells appeared to correlate with pluripotency and neurodevelopment^5,7,12–16^. Moreover, 5hmC modifications co-localize with gene bodies and enhancers are known to mark for transcription activation^6,17,18^. Notably, in several recent studies, the 5hmC-modified loci have been shown to serve as informative biomarkers for a variety of human cancers and other complex diseases^19–26^. Interestingly, two early studies that investigated the 5hmC in circulating cell-free DNA from patients with solid tumors suggested that 5hmC had the potential to be specific biomarkers for human cancers arising from different tissue origins^19,20^.

Despite a plethora of studies that link changes in global 5hmC with disparate developmental processes as well as pathobiology (e.g., cancer initiation and progression), the knowledge of the precise functions of 5hmC remains incomplete. We reasoned that a map of genome-wide 5hmC distributions across different human tissue types would provide an opportunity to enhance our understanding of the 5hmC regulatory control in normal tissues and dysregulation implicated in human diseases. Previous studies have either used microarray-based techniques^27,28^ which lack genome-wide coverage or only focused on limited tissue types^27–29^. In this study, we report an extensive 5hmC human tissue map obtained using 5hmC-Seal, a sensitive chemical labeling and pull-down method, followed by next-generation sequencing (NGS)^6,30^. Specifically, a map of 5hmC was systematically defined and characterized across 19 tissue types derived from ten organ systems from donors of European ancestry. The 5hmC-enriched regions were further evaluated for their regulatory potential and tissue specificity by comparing with gene expression data and the *cis*-regulatory element data from the Roadmap Epigenomics Project^31^.

## Results

### Genome-wide profiling of 5hmC across major human organs and tissue types

**Fig. 1:**
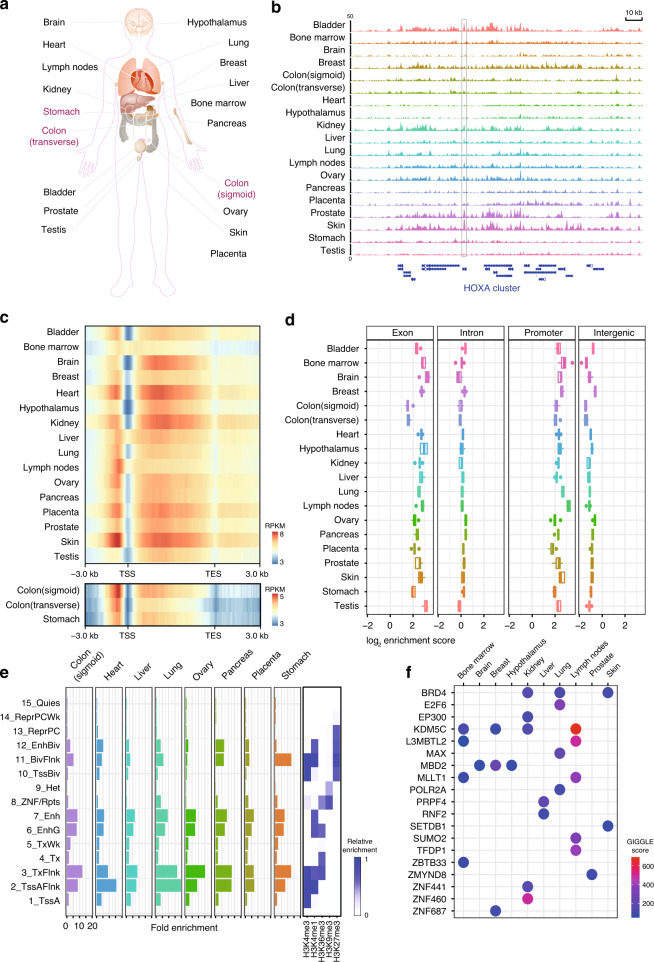
Landscape of 5hmC across different tissues. **a** Schematic plot showing all the organ tissues analyzed in this study. Tumor adjacent tissues are marked in red. **b** Genome Browser view depicting 5hmC genomic distributions at the HOXA gene cluster for one representative donor’s profile for each of the 19 tissue types assayed. The peak outlined with a box shows a highly variable region as an example. **c** Metagene plot of 5hmC profiles across different tissues showing underrepresentation of 5hmC at TSS regions and over-representation at gene bodies and promoters. The color ranges indicate RPKM values. Upper panel, normal tissues; lower panel, tumor adjacent tissues. TSS, transcription start site. TES, transcription end site. **d** Boxplots showing genomic enrichment of 5hmC peaks at RefSeq exons, introns, promoters, and intergenic regions. *N* = 5 biologically independent samples were used (*n* = 4 for hypothalamus and *n* = 6 for sigmoid and transverse colon). For all boxplots, center line represents median, bounds of box represent 25th and 75th percentiles and whiskers are Tukey whiskers. **e** Enrichment of 5hmC peaks on 15 chromHMM chromatin states from the Roadmap Epigenomics Project for 8 tissue types. Histone modification emissions data are directly from the Roadmap Epigenomics Project. **f** Enrichment of trans-acting factors binding sites on 5hmC peaks in different tissues. Higher GIGGLE score means higher possibility of enrichment.

To construct an extensive map of genome-wide 5hmC modifications, we collected fresh-frozen tissue samples representing 19 tissue types from ten major organ systems: the nervous, cardiovascular, digestive, reproductive, endocrine, respiratory, urinary, integumentary, skeletal, and lymphatic systems. The tissue samples were obtained by autopsy or surgery from five individual donors for each type, except that four donors contributed to the hypothalamus and six donors to the sigmoid and transverse colon tissue (Fig. 1a and Supplementary Data 1). Of the total of 96 specimens, 79 samples were taken from non-cancerous organs, while the remaining 17 were from normal adjacent tissues upon tumor resection, including sigmoid colon, transverse colon, and stomach samples. All the tissue donors were adults of European ancestry, and 48% were males, with an average age of 52 years. In total, 5hmC-Seal libraries were generated and sequenced for 96 samples with an average of ~42 million paired-end reads per sample. The unique mapping ratio was consistent across all samples (56.11% on average), with a low duplication level (7.69% on average), indicating a high-quality 5hmC enrichment dataset from the 5hmC-Seal profiling.

A previous report using tiling microarrays suggested that the HOXA gene cluster had highly variable 5hmC levels in different tissue types^27^. We thereby investigated whether this observation could be recapitulated in our 5hmC-Seal datasets between tissue types. Indeed, the location and enrichment levels of peaks vary across tissue types, especially between distantly-related tissues (e.g., brain vs. kidney, shown in Fig. 1b). On the other hand, the enrichment of 5hmC at the HOXA gene cluster appeared to be highly consistent between donor samples within the same tissue type (Supplementary Fig. 1a).

Compared with single-base resolution methods, i.e., TAB-seq and oxBS-seq that provide high-resolution 5hmC mapping^32^, a high correlation (Spearman *r* = 0.82) was observed between our dataset and the publicly available single-base resolution 5hmC maps^33^ (Supplementary Fig. 1b). Comparisons between our 5hmC-Seal signals and the local CpG density revealed no correlations (Supplementary Fig. 1c). Taken together, these data confirmed the profiling accuracy and reproducibility of the genome-wide 5hmC profiles we obtained in various tissues, which provided a unique resource to study tissue-specific distributions of 5hmC in the human genome.

### Common features of the 5hmC distributions in various human tissues

Having established a dataset of genome-wide 5hmC distributions that could differentiate tissue identities, we next examined the distributions of 5hmC across functional regions to identify shared features in 5hmC deposition across different tissues. It has been established that the 5hmC is deficient at transcription start sites (TSSs) and enriched at promoters and gene bodies in mammalian genomes^5,6^. The metagene plots of our 5hmC profiles demonstrated the expected relative distribution patterns of 5hmC at TSSs, promoters, and gene bodies for each of the 19 tissue types (Fig. 1c). Although the 5hmC signals from colon and stomach tissues showed a trend of lower levels relative to other tissues, potentially due to tissue-specific characteristics or epigenomic changes caused by the presence of tumors in these donors, the general patterns of 5hmC distribution are still consistent (Fig. 1c).

The distributions and densities of 5hmC modifications across the human genome were also identified and analyzed using the 5hmC peaks called by MACS2. Although the total number of the identified 5hmC peaks per sample ranged from 4,302 to 193,580 (FDR < 0.05), within each tissue type the number of peaks per donor sample was similar (Supplementary Fig. 2a). For example, the lowest peak numbers were obtained from bone marrow samples (12,976 on average), while the highest peak numbers were obtained from the placenta samples (116,834 on average). The peaks from each sample were high-quality subsets of peaks called from merging samples for each tissue type (Supplementary Fig. 2b, c). Upon examination of different genomic regions (i.e., exons, introns, promoters, and intergenic regions), the 5hmC peaks were found to be overrepresented at exons and promoters, while underrepresented at intergenic regions (Fig. 1d), demonstrating that in spite of different tissue identities, the 5hmC loci were consistently distributed within known genomic locations of 5hmC^6^. Intriguingly, despite the relatively low number of called peaks, the bone marrow 5hmC peaks still revealed high enrichment at promoters, suggesting a tight regulation of the genomic distribution of 5hmC. In summary, the fact that 5hmC is preferentially distributed across genic regions, rather than intergenic regions, supports the hypothesis that 5hmC is a mark of active transcription and gene activation.

The 5hmC distributions also flanked transcription factor binding sites at promoters. In particular, genomic 5hmC peak loci showed increased occupancy in regions of evolutionarily conserved elements compared to controls of randomly shuffled peak regions in the genome (Supplementary Fig. 2d). Not surprisingly, the CG motif was the most significant motif across the 5hmC peaks for all tissues (Supplementary Fig. 2e), indicating importantly the specificity and precision of the 5hmC-Seal pull-down assay.

Moreover, the t-SNE clustering of 5hmC distributions within peaks separated different tissue types, whereas intra-tissue samples (i.e., samples from one tissue type from different donors) were clustered together (Supplementary Fig. 2f). For example, the 5hmC profiles of the prostate (five individuals) were clustered as a single group, and the prostate group was clustered distinctively from the heart tissue samples. This observation suggested that 5hmC profiles showed higher inter-tissue than inter-individual variability. Furthermore, the proximity between clusters in the t-SNE space correlated with the closeness of tissues that are functionally and developmentally more related (Supplementary Fig. 2f). For example, the brain and hypothalamus samples were clustered closest, reflecting that the 5hmC distributions of the central nervous system (CNS)-related tissues had the smallest variability. The similar observation was also apparent in the gastrointestinal system (i.e., colon and stomach samples).

### 5hmC at active genomic regions

The human genome can be divided into different functional segments based on various epigenomic modifications^34^. We next sought to investigate 5hmC modifications in the context of different epigenomic regions by comparing with publicly available epigenomic data. The ENCODE project includes five histone modifications (i.e., H3K4me3, H3K4me1, H3K36me3, H3K9me3, and H3K27me3) that separate the genome into 15 different chromatin states^34^. We targeted eight representative tissues (i.e., sigmoid colon, heart, liver, lung, ovary, pancreas, placenta, and stomach) to evaluate the 5hmC distributions and chromatin states. We found that 5hmC showed similar distribution patterns in all eight tissue types, highly enriched in regulatory chromatin states mainly marked by H3K4me1 (Fig. 1e) and defining active and flanking transcription start sites (i.e., TssAFlnk and TxFlnk, respectively), enhancer regions (enhancers—Enh and genic enhancers—EnhG) and also bivalent enhancer regions (EnhBiv—bivalent enhancer and BivFlnk—bivalent enhancer and TSS flanking region), the latter of which were reported to have functions in embryonic development and lineage specification^5^ (Fig. 1e).

Considering the interplay between 5hmC and 5mC modifications for transcriptional regulation, we re-analyzed the whole-genome bisulfite sequencing data (WGBS) for seven representative tissues (i.e., sigmoid colon, heart, liver, lung, ovary, pancreas, and stomach) available from the Roadmap Epigenomics Project^31^. By comparing the methylation levels of 5hmC loci, we determined the median methylation levels in the 5hmC peaks ranging from 0.78 for the ovary to 0.87 for the liver (Supplementary Fig. 3a), consistent with the notion that 5hmC is the next intermediate product in the 5mC demethylation pathway. To determine the 5hmC modification at different genomic segments based on the cytosine methylation context, we analyzed DNA methylation canyons (>3.5 kb unmethylated regions), control unmethylated regions (cUMR, between 1 and 3.5 kb), partially methylated domains (PMD, hundreds of kb regions with relatively high methylation levels), and lowly methylated regions (LMR, <30 CpGs per few hundred-to-thousand bp)^35^. While the UMRs and LMRs correspond to proximal and distal regulatory regions, the PMDs always represent a transcriptionally repressed state. Consistent with previous reports^30^, our 5hmC dataset showed that 5hmC was preferentially enriched at the borders of methylation canyons and the cUMRs, instead of being localized at the center of these regions (Supplementary Fig. 3b), supporting the idea of active cytosine demethylation at these regions. In contrast, the 5hmC loci were rarely found at borders or centers of the PMDs, suggesting little to no active demethylation at these heterochromatin regions. Last, high 5hmC abundance was observed at the LMR centers, indicating the enrichment of 5hmC on distal regulatory sites (Supplementary Fig. 3b).

We also investigated the subset of 5hmC peaks annotated to known trans-acting factor binding sites in various tissue types. Transcriptional and epigenomic regulators such as BRD4, EP300, EZH2, TFDP1, and KDM5C were among the top-ranking enriched factors, suggesting sequential regulation from demethylation to final transcriptional activation (Fig. 1f), consistent with recent studies that revealed 5hmC to be a communication hub in the chromatin network of embryonic stem cells^36^. Collectively, these data suggest that 5hmC is preferentially distributed at active genomic regions with potential implications in transcriptional regulation.

### Tissue-specific 5hmC signatures mark tissue-specific functional genes

**Fig. 2:**
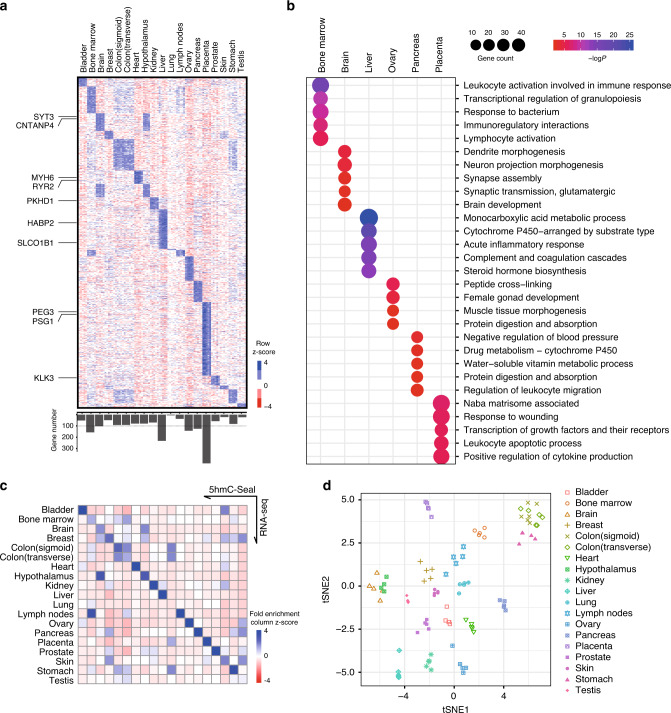
Tissue-specific, 5hmC-enriched genes are associated with highly tissue-specific functions. **a** Relative 5hmC-modification levels on tissue-specific 5hmC-enriched genes in each tissue type. Rows are distinct 5hmC-modified genes. Higher z score indicates enrichment; lower z score indicate depletion of 5hmC signal. Lower panel, numbers of tissue-specific 5hmC-enriched genes for each tissue type. Dashed line represents 100. **b** Functional enrichment for tissue-specific 5hmC-enriched genes. Only tissues with over 100 tissue-specific 5hmC-enriched genes are shown. Top 5 enriched functional terms are ranked by enrichment significance. **c** Heatmap showing fold enrichment of tissue-specific 5hmC-modified genes with tissue-specific expressed genes from accompanying RNA-Seq data. **d** t-SNE clustering of genomic 5hmC distributions on exons for all donor tissue samples. Colored symbols indicate the organ/tissue associated with each 5hmC profile.

We next asked whether the 5hmC modifications marked tissue-specific genes. We employed the definition used by the Human Protein Atlas Project (HPA)^37^ with modified parameters (i.e., fold change cutoff = 2; RPKM cutoff = 10) to identify tissue-specific 5hmC-modified genes. A total of 1,723 tissue-specific 5hmC-modified genes were detected that separate all tissues (Fig. 2a), with the placenta showing the highest number of tissue-specific genes. For those samples from related anatomical or physiological systems, an overlap of the direction of 5hmC enrichment (i.e., enriched vs. depleted 5hmC) was apparent (Supplementary Fig. 4a). For example, the brain-specific 5hmC-modified genes also showed higher modification levels in the hypothalamus samples, compared to other tissue types. In addition, transverse and sigmoid colon segments exhibited similar modification levels for tissue-specific genes, highlighting their relatedness, despite arising from different embryonic lineages (Fig. 2a). Also evident were those genes shared between bone marrow and lymph nodes as is expected because of their association as part of the lymphatic system. We also identified gene pathways based on tissue-specific, 5hmC-modified genes, and found that these pathways reflected tissue-specific functions. For example, using the Metascape-based functional enrichment analysis^38^, the top-ranking enriched functional clusters for the brain-specific 5hmC genes were highly CNS-specific, including dendrite morphogenesis, neuron projection morphogenesis, and synapse organization (Fig. 2b, Supplementary Data 2). These clearly implicating trends between detected pathways and tissue types were also found in the bone marrow, liver, ovary, pancreas, and placenta samples with over 100 tissue-specific, 5hmC-modified genes detected (Fig. 2b).

Next, using the dataset from HPA, we observed a 30.7-fold enrichment for pancreas-specific, 5hmC-modified genes in pancreas-specific transcripts from the HPA, compared with other tissue-specific, 5hmC-modified genes (Supplementary Fig. 4b), indicating an extremely high correlation of 5hmC density and transcription in tissue-specific genes. Indeed, this same observation was apparent for most tissue types matched (Supplementary Fig. 4b), only with a few exceptions likely due to a low number of tissue-specific 5hmC-modified genes identified or the lack of closely matched tissue types within available HPA data (Supplementary Fig. 4b).

We also generated gene expression profiles by RNA-seq from the same 96 samples used for the 5hmC-Seal profiling. Similar to what shows in the 5hmC-based clustering, gene expression for all RefSeq genes separated different tissue types clearly (Supplementary Fig. 4c). We used the HPA algorithm with the same parameters (i.e., fold change cutoff = 4, RPKM cutoff = 1) and identified a total of 7,978 tissue-specific expressed genes. Consistently, our tissue-specific expressed genes were shown to be enriched with the tissue-specific genes for the same tissue types from the HPA (Supplementary Fig. 4d), indicating the reliability of our RNA-seq dataset. Specifically, all of our RNA-seq results, except for the lung and testis samples that had few tissue-specific 5hmC-modified genes (6 for lung and 23 for testis), showed high consistency between tissue-specific 5hmC and gene expression (Fig. 2c). Notably, gene-level 5hmC distributions can readily separate different tissue types (Fig. 2d).

### 5hmC is enriched on tissue-specific genes

**Fig. 3:**
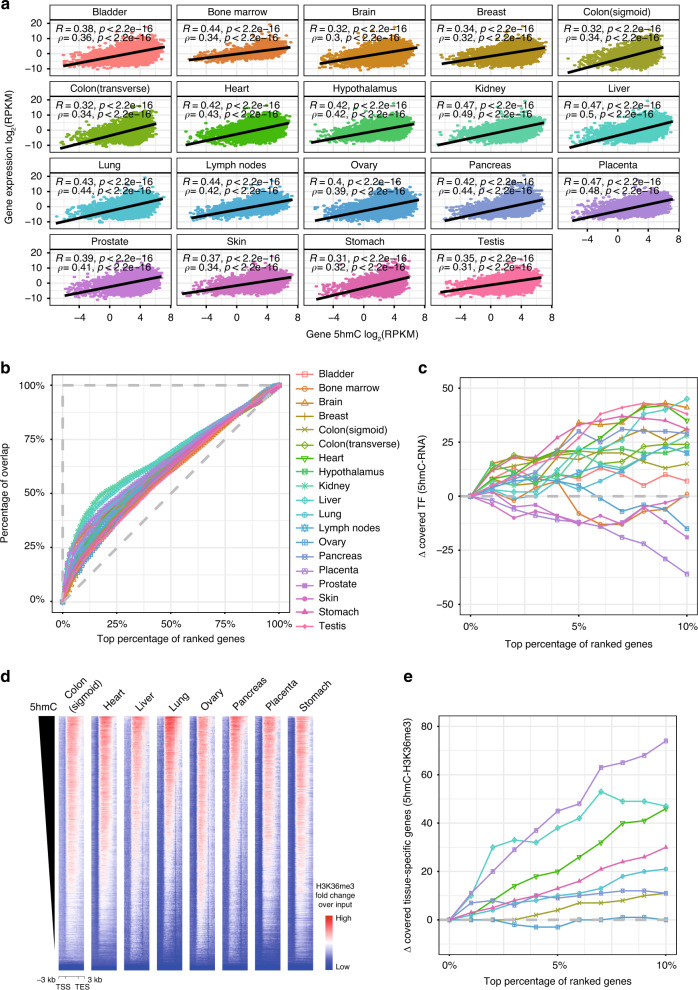
Enrichment of 5hmC on tissue-specific genes. **a** Scatter plots showing correlation of gene-body 5hmC levels with gene expression levels. *R* represents the Pearson correlation coefficient, and *ρ* represents the Spearman correlation coefficient. **b** Correspondence at the top (CAT) plot showing percentages of gene overlap against top percentages of 5hmC-modified genes and expressed genes. Diagonal dashed line represents results from two random gene sets, and the horizontal dashed line represents results from two exact gene sets. **c** Subtractions of covered transcription factors for top 10% 5hmC-modified genes and top 10% highly expressed genes. **d** Heatmaps showing correlation of gene-body 5hmC levels with gene-body H3K36me3 levels. TSS, transcription start site. TES, transcription end site. **e** Subtractions of covered tissue-specific expressed genes for top 10% 5hmC-modified genes and top 10% H3K36me3-modified genes.

We then asked to what extent 5hmC modification could be a surrogate for gene expression. In all tissues, we observed a moderate positive correlation of the levels between gene-body 5hmC and the corresponding transcripts (Fig. 3a), suggesting that the gene-level 5hmC modifications can reflect the gene expression status in different human tissues.

We also noticed that highly 5hmC-modified genes tend to have a medium level of gene expression. In particular, we performed a correspondence at the top (CAT) analysis (Fig. 3b). Genes were ranked based on gene expression level and 5hmC-modification level, respectively, and the proportions of genes in common were plotted against different proportions of ranked genes. Intriguingly, we observed higher slopes at the left end, suggesting greater relative concordance changes and less absolute concordance among the top genes (Fig. 3b). These observations further suggest that while gene-body 5hmC is a faithful mark to indicate gene expression, the absolute 5hmC level in gene-body itself is not sufficient for accurately predicting the gene expression level for the most highly expressed genes.

After examining the gene categories of highly 5hmC-modified genes and highly expressed genes, we found that more transcription factors are represented in the top 1–10% 5hmC-modified gene set compared to the top expressed gene set (Fig. 3c), suggesting more regulatory and dynamic functions of the 5hmC-modified genes. It is known that transcription factors are important gene regulation components, and are often differentially expressed in different tissues^37^. Consistently, the comparison of tissue-specific genes defined by 5hmC modification and gene expression revealed a higher proportion of transcription factors in tissue-specific, 5hmC-modified genes relative to tissue-specific expressed genes alone (Supplementary Fig. 5a, Supplementary Data 3). This suggested that the 5hmC modifications might have occurred in genes that were undergoing an active process of expression regulation through transcriptional modulation rather than those genes whose transcription was well established.

We next examined whether a model could be built to classify a tissue’s identity using genome-wide 5hmC profiles or transcriptomes. We generated multinomial logistic regression models, with the range of predicted probability revealing the highest score for each tissue using the cognate models for that tissue (Supplementary Fig. 5b). Significant off-diagonal probability scores were detected in the two types of colon tissue (sigmoid and transversal). This exception is not entirely unexpected, considering the overlap of the sigmoid and transverse colon 5hmC genomic distribution clusters (Supplementary Fig. 2f), and that a previous study showed that <100 differentially expressed genes separated these two colon tissue types during embryonic development^39^. We observed many more off-diagonal, non-cognate tissue predictions for transcriptome-based classification. Some of these classification pairs are similar to those observed using the 5hmC data (i.e., sigmoid colon and transverse colon, or brain and hypothalamus), but some could not be directly explained by tissue relatedness (e.g., heart and breast, hypothalamus and skin, or brain and ovary). Taken together, these data suggest that 5hmC profiles could be more relevant than gene expression profiles for genes undergoing active transcriptional regulation.

We further focused on tissue-specific differentially methylated regions (DMRs) defined by the Roadmap Epigenomics project. We suspect that at lowly methylated DMRs many 5mCs are under oxidation and demethylation, which potentially gives rise to relatively high 5hmC levels or relatively high density of 5hmC. Therefore, tissue-specific lowly methylated DMRs at promoters would result in high 5hmC deposition in related gene bodies that are also tissue specific. Indeed, the 5hmC signal was highly abundant on tissue-specific genes defined by lowly methylated DMRs at their promoters in the liver and pancreas, respectively. In concert, the 5hmC density was lower at the same gene sets in other tissue types (Supplementary Fig. 5d), consistent with the relation between active demethylation and 5hmC deposition.

We next explored whether similar conclusions could be obtained from comparisons of 5hmC profiles and H3K36me3 signals on gene bodies. High consistency was observed in all eight representative tissues (Fig. 3d). The CAT curves showed similar patterns as those for the top 5hmC-modified genes and the most highly expressed genes (Supplementary Fig. 5c), although differences also exist between these two modifications. The top 5hmC-modified genes were found to contain more tissue-specific expressed genes than the top H3K36me3-modified genes (Fig. 3e). Therefore, compared to the transcriptome and H3K36me3 distribution, the 5hmC profiles are more enriched on tissue-specific genes, thus supporting gene-body 5hmC modification as a promising surrogate for tissue-specific biomarkers during tissue development and disease pathogenesis.

### 5hmC is enriched on tissue-specific enhancers

**Fig. 4:**
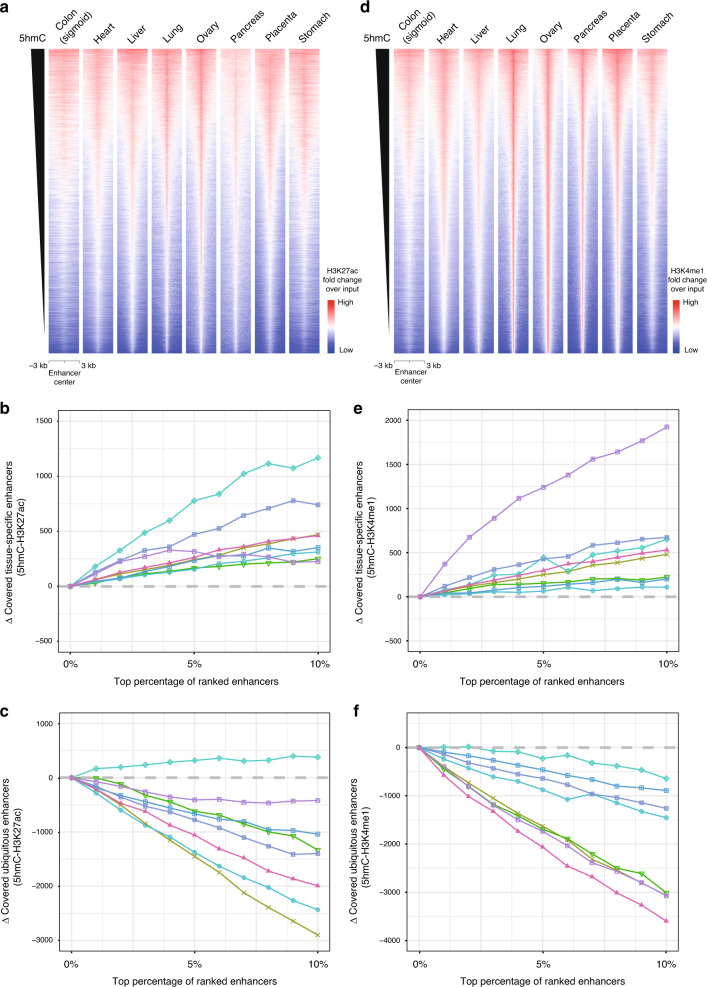
Enrichment of 5hmC on tissue-specific enhancers. **a** Heatmaps showing correlation of 5hmC and H3K27ac signals at all enhancers in 8 representative tissue types. **b** Subtractions of covered tissue-specific enhancers for the top 10% 5hmC-modified enhancers and the top 10% H3K27ac-modified enhancers. **c** Subtractions of covered ubiquitous enhancers for the top 10% 5hmC-modified enhancers and the top 10% H3K27ac-modified enhancers. **d** Heatmaps showing correlation of 5hmC and H3K4me1 signals at all enhancers in 8 representative tissue types. **e** Subtractions of covered tissue-specific enhancers for the top 10% 5hmC-modified enhancers and the top 10% H3K4me1-modified enhancers. **f** Subtractions of covered ubiquitous enhancers for the top 10% 5hmC-modified enhancers and the top 10% H3K4me1-modified enhancers.

We next systematically assessed 5hmC-modification status across all enhancer regions as defined by the Roadmap Project for the eight representative tissues (Fig. 4). The whole-genome profiling results showed that the 5hmC profiles were highly associated with the activation marker H3K27ac (Fig. 4a and Supplementary Fig. 6). Consistent with our gene-level study, the top 5hmC-modified enhancers were found to be enriched in more tissue-specific enhancers and less ubiquitous enhancers than the top H3K27ac-modified enhancers (Fig. 4b, c). Notably, the liver samples had higher 5hmC signals on both tissue-specific enhancers and ubiquitous enhancers, potentially due to presence of high total methylation levels at the 5hmC peak regions (Supplementary Fig. 3a).

Furthermore, for H3K4me1, our analysis showed that high 5hmC-modified enhancers tend to possess high H3K4me1 modification levels for the eight tissue types (Fig. 4d). The top 5hmC-modified enhancers had more tissue-specific enhancers and less ubiquitous enhancers than the top H3K4me1-modified enhancers (Fig. 4e, f). Taken together, our results showed that 5hmC is more enriched on tissue-specific enhancers than classical enhancers marked by H3K27ac and H3K4me1, suggesting the potential of 5hmC as a better tissue-specific biomarker for tissue-type classification.

## Discussion

We provide here a detailed map of active DNA 5mC oxidation via direct detection of 5hmC across 19 different human tissues representing ten different organ systems. Compared to previous studies^27–29^, our 5hmC-Seal data present genome-wide profiles of cytosine hydroxymethylation in DNA across broad-scale tissue types; this extensive map enabled us to uncover tissue-specific differences in 5hmC deposition and regulation. We found that 5hmC is preferentially enriched at tissue-specific genes and regulatory elements, regardless of the tissue or organ type involved. High enrichment of tissue-specific 5hmC signals for most human tissues was observed, revealing a strong correlation of 5hmC deposition and transcription of tissue-specific genes in real human tissues. This tissue specificity of transcription was observed in a manner consistent with the known biology of tissue and organ systems. Enriched transcription factor binding sites under 5hmC peaks in different tissues may facilitate future investigation of orchestrated tissue-specific developmental regulation processes. Considering that 5hmC deposition is catalyzed by TET enzymes, our observations support the notion that TETs play a pivotal role in restructuring the 5mC landscape during the development of specific tissues^12,14,15^. Correspondingly, although DNA methylation levels are highly consistent across different tissue types, 5hmC levels were found to vary significantly between tissues^27^.

Previous studies have shown that 5hmC-modified loci can serve as excellent biomarkers for the diagnosis and prognosis of human diseases including cancer^19–21,23–26^. These data suggest that 5hmC may mark specific genes or promoter/enhancers in disease samples that undergo concerted changes representative of the disease pathogenesis in question. The human tissue 5hmC map presented herein provides a critical and valuable resource to integrate with known data of 5mC distribution, chromatin accessibility, and various histone marks in human tissues to further understand the role of epigenomic regulation in human development and disease progression.

In conclusion, we report here a detailed map of 5hmC profiles across a variety of human tissue types using 5hmC-Seal, a technology being developed for clinical applications. We confirm the important roles of the TET-catalyzed 5mC oxidation at tissue-specific genes and enhancers in non-disease human systems across 19 discrete tissue types examined. We provide a resource of the comprehensive 5hmC distribution but also tissue-specific, 5hmC-modified regions in major human tissues. We expect that the data reported here will significantly facilitate further understanding of human development and disease progression as well as the clinical applications of 5hmC as novel epigenetic biomarkers.

## Methods

### Tissue collection

Fresh-frozen tissue specimens were acquired from Proteogenex Inc., either obtained through patient surgery or cadaver-sourced from 19 tissue types, each having five individual donors; no tissue preservatives were utilized in this study. Donors were 50–70 years of age and balanced across sexes for all relevant tissues. Either donors or next of kin were consented for DNA, RNA, and protein analysis through an ethics board approved study protocol at Proteogenex. Death of individuals was generally due to accident or cause unrelated to the tissue collected and post-mortem interval was <6 h. Fresh-frozen tissue was aliquoted into two to three pieces, depending on weight available, and stored at −80 °C until DNA extraction. Thirty milligrams of tissue samples were homogenized using the GentleMACS dissociator (Milteyni Biotec) according to manufacturer directions. Total genomic DNA was extracted from the resulting homogenate using the DNeasy Blood & Tissue Kit (QIAGEN) according to manufacturer directions. DNA eluates were quantified using a Nanodrop microvolume spectrophotometer (ThermoFisher), aliquoted in 100 ng quantities, and stored at −20 °C until use. All DNA aliquot concentrations were re-quantified using the Qubit dsDNA high sensitivity kit (ThermoFisher) prior to library construction.

### 5hmC-Seal library construction

The 5hmC-Seal was performed as previously described in Han et al.^30^. Briefly, 100 ng genomic DNA were fragmented in Tagmentation buffer at 55 °C. Fragmented DNA was purified by Zymo DNA Clean and Concentration Kit. Then, the selective 5hmC chemical labeling was performed in glucosylation buffer (50 mM HEPES buffer pH 8.0, 25 mM MgCl_2_) containing above fragmented DNA, βGT, N3-UDP-Glc, and incubated at 37 °C for 2 h. After DNA purification in ddH_2_O, DBCO-PEG4-Biotin (Click Chemistry Tools) was added and incubated at 37 °C for 2 h. The biotin-labeled DNA was pulled down by C1 Streptavidin beads (Life Technologies) for 15 min at room temperature. Next, the captured DNA fragments were subjected to PCR amplification using Nextera DNA sample preparation kit. The resulting amplified product was purified by 1.0X AMPure XP beads. Input library was made by direct PCR from fragmented DNA directly without labeling and pull-down. The libraries were quantified by a Qubit fluorometer (Life Technologies) and sequenced on NextSeq 500.

### RNA-seq library construction

RNA-seq libraries were constructed from 1 μg of total RNA after ribosomal depletion following the manufacturer’s protocols (Illumina). Briefly, the purified RNA was fragmented, reverse transcribed into first-strand cDNA using random primers. This is followed by second-strand cDNA synthesis using RNase H and DNA Polymerase I. Adaptor was ligated after a single “A” was added. Final cDNA libraries were created by purification, enrichment with PCR, size validated using Agilent Bioanalyzer, and concentration validated by qPCR (Kapa Biosystems/Roche). All libraries were normalized to 10 nM and pooled together, denatured with 0.2 N NaOH, and diluted to a final concentration of 1.4 pM. Finally, 1.3 ml of 1.4 pM pooled libraries were sequenced on NextSeq 500 generating 25 million 75 bp paired-end reads per sample.

### 5hmC-Seal data analysis

Paired-end reads were first trimmed by Trim_Galore (https://github.com/FelixKrueger/TrimGalore) to remove adaptor sequences and low-quality nucleotides. High-quality reads were then aligned to hg19 reference genome by Bowtie, with uniquely mapped reads kept for all downstream analyses. PCR duplicates were depleted by Samtools software and further normalization was performed with Deeptools using RPKM normalization strategy. Metagene profiles were generated based on normalized signals by Deeptools. Bioconductor package ChIPQC was used for quality control of all 5hmC-Seal libraries. MACS2 was used to call 5hmC peaks and we also excluded all peaks located in ENCODE blacklist regions. Annotation and enrichment analysis of 5hmC peaks were performed with Homer toolkit. Promoters were defined as regions from −1 kb to +100 bp of TSS by Homer. Reads were counted either on whole exons for the comparisons of RNA-seq and H3K36me3 or on enhancers for the comparisons of H3K27ac and H3K4me1 by FeatureCounts.

### Comparisons with public single-base resolution 5hmC data

To compare our enrichment-based 5hmC profiles with single-base resolution 5hmC maps, we downloaded public TAB-seq dataset on prostate from GSE104780. The TAB-seq and 5hmC-Seal signals were averaged on 200-kb sliding windows across the genome.

### Tissue-specific 5hmC-modified genes

To systematically identify tissue-specific 5hmC-modified genes and compare with Human Protein Atlas, we employed the same strategy on gene-level reads counts. Basically, HPA classifies three groups of tissue-specific expressed genes according to stringency of tissue specificity: The highest stringency is called “tissue enriched”, meaning tissue gene expression level at least fourfold higher than expression levels in all 37 tissues in HPA. The next highest specificity is called “tissue enhanced”, this group of tissue gene expression is at least fourfold higher than the average expression levels in all other tissues. Finally, the lowest tissue specificity of genes is called “group enriched”, which are genes that are expressed in a group of related tissues (comprising 2–5 tissues) and average expression level in this group is at least fourfold higher than all other tissues.

Herein, tissue enriched 5hmC-modified genes are defined to have at least twofold higher modification level in a particular tissue compared to any other tissues, tissue enhanced 5hmC-modified genes are those having at least twofold higher modification level in a particular tissue compared to the average level in all other tissues, and group enriched 5hmC-modified genes are defined to have at least twofold higher average modification level in a group of 2–7 tissues compared to any other tissue. We only considered “tissue enriched” and “tissue enhanced” groups as tissue-specific, 5hmC-modified genes to exclude impacts from potential epigenomic changes of normal adjacent tissues (colon and stomach).

### RNA-Seq data analysis

Paired-end reads were first trimmed with TrimGalore (https://github.com/FelixKrueger/TrimGalore) to remove adaptor sequences and low-quality nucleotides. High-quality reads were then aligned to hg19 reference genome by HISAT2, with uniquely mapped reads kept for all downstream analyses. R package edgeR was used to calculate RPKM values.

### Multinomial logistic regression

We used elastic net in R package glmnet for the 5hmC-Seal and RNA-seq multinomial logistic regression analysis based on gene-body reads count. For the 5hmC-Seal data, weakly represented genes with CPM (counts per million) >3 in <5 samples were excluded from analysis. Before any fitting, CPM was log2 transformed, genes were filtered to include the top 65% of the most variable genes for model fitting task. Hyper-parameters of the regularization model were selected based on out-of-fold performance on five repetitions of fourfold cross-validated analysis of the training data. Out-of-fold assessments are based on the samples in the left-out fold at each step of the cross-validated analysis. We used the same method for RNA-seq dataset, except that weekly expressed genes were defined as CPM > 2 in <5 samples.

### Integration with WGBS data

Genomic Segments based on WGBS data were downloaded from GSE113405. Basically, different epigenomes from the Roadmap project were used to search DNA methylation Canyons, control UMRs (cUMRs), Partially Methylated Domains (PMD), and Lowly Methylated Regions (LMRs) by MethylSeekR package^40^. Distributions of 5hmC signals across different genomic segments were generated by Deeptools.

### Integration with histone modification data

Chromatin states data based on five histone modifications (H3K4me1, H3K4me3, H3K9me3, H3K27me3, and H3K36me3) were downloaded from the Roadmap project. Overlap between 5hmC peaks and different chromatin states was conducted by Bedtools. Ubiquitous and tissue-specific enhancers were downloaded from the Roadmap DNaseI dataset.

### Reporting summary

Further information on research design is available in the Nature Research Reporting Summary linked to this article.

## Supplementary information

Supplementary Information

Supplementary Data 1

Supplementary Data 2

Supplementary Data 3

Description of Additional Supplementary Files

Reporting Summary

## Data Availability

The raw and processed 5hmC-Seal and RNA-seq data have been deposited into NCBI Gene Expression Omnibus (GEO) database with accession number ‘GSE144530’. Public TAB-seq data used in this study were downloaded from ‘GSE104780’. Genomic Segments dataset based on WGBS data was downloaded from ‘GSE113405’. Chromatin states and enhancers data were directly downloaded from Roadmap Epigenomics Project website (http://www.roadmapepigenomics.org).
