# Supplementary material for: A human tissue map of 5-hydroxymethylcytosines exhibits tissue specificity through gene and enhancer modulation
Source: Nat Commun. 2020 Dec 2;11:6161. doi: 10.1038/s41467-020-20001-w (PMC7710742; doi:10.1038/s41467-020-20001-w)
Supplement: Supplementary file 1 — Supplementary Information [file 41467_2020_20001_MOESM1_ESM.pdf]

Supplementary Information

A human tissue map of 5-hydroxymethylcytosine exhibits tissue specificity through gene and enhancer modulation

Xiao-Long Cui[1,2,7], Ji Nie[1,2,7], Jeremy Ku[3,7], Urszula Dougherty[4], Diana C. West-Szymanski[2,4], Francois Collin[3], Christopher K. Ellison[3], Laura Sieh[1,2], Yuhong Ning[3], Zifeng Deng[4], Carolyn W.T. Zhao[1,2], Anna Bergamaschi[3], Joel Pekow[4], Jiangbo Wei[1,2], Alana V. Beadell[1,2], Zhou Zhang[5], Geeta Sharma[6], Raman Talwar[3], Patrick Arensdorf[3], Jason Karpus[1,2], Ajay Goel[6], Marc Bissonnette[4], Wei Zhang[5], Samuel Levy[3], Chuan He[1,2,*]

[1]Department of Chemistry, Department of Biochemistry and Molecular Biology, Institute for Biophysical Dynamics, University of Chicago, Chicago, IL, USA

[2]Howard Hughes Medical Institute, University of Chicago, Chicago, IL, USA

[3]Bluestar Genomics Inc., San Diego, CA, USA

[4]Department of Medicine, University of Chicago, Chicago, IL, USA

[5]Department of Preventive Medicine, Northwestern University Feinberg School of Medicine, Chicago, IL, USA

[6]City of Hope Comprehensive Cancer Center, Duarte, CA, USA

[7]These authors contributed equally to this work

[*]Correspondence: chuanhe@uchicago.edu (C.H.)

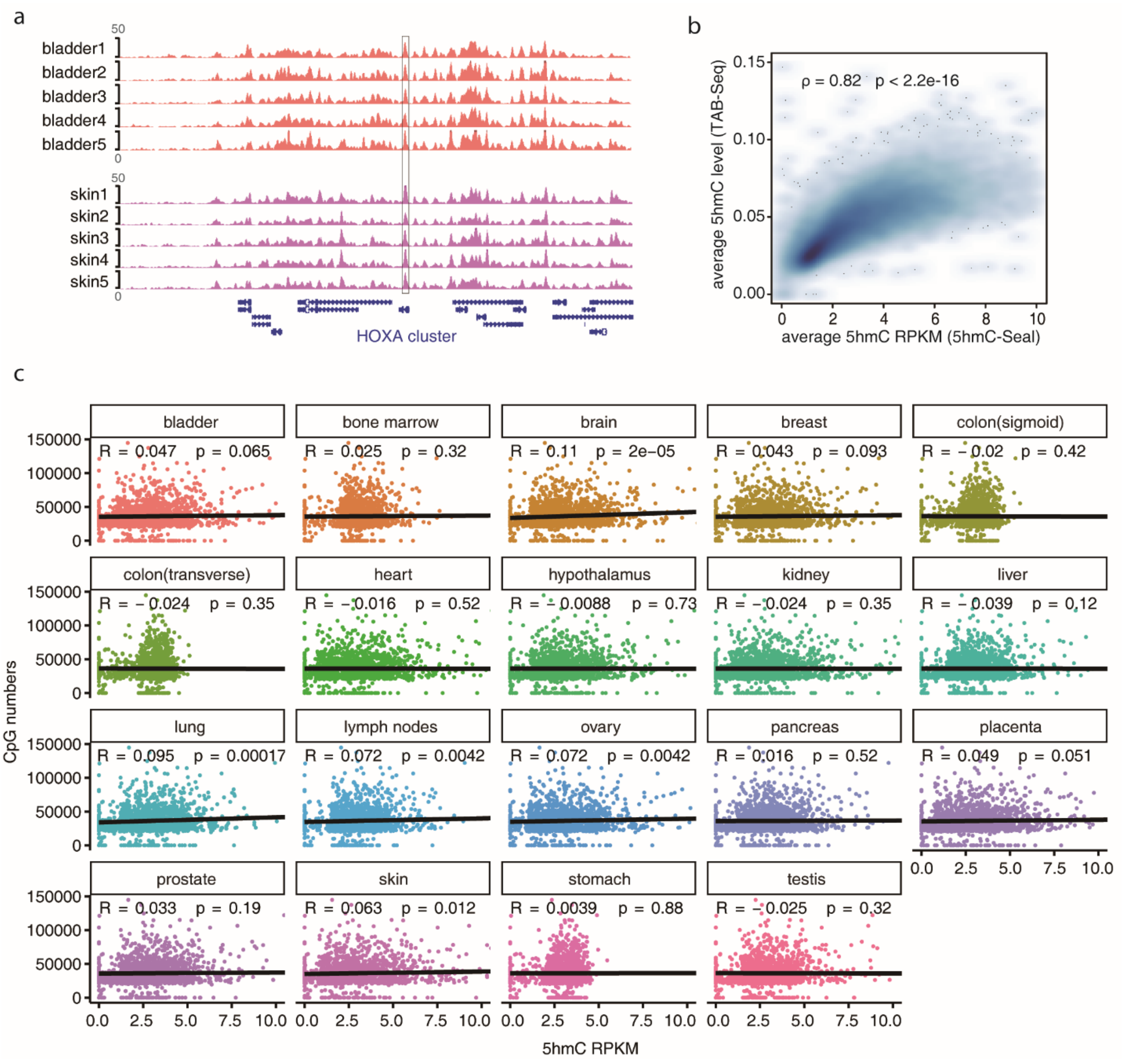

Supplementary Figure 1. Quality control of 5hmC-Seal profiles. a, Genome browser view of 5hmC genomic distributions at the HOXA gene cluster for each donor of bladder tissue (n = 5) and skin tissue (n = 5). **b**, Density plot showing correlations of 5hmC-Seal data with TAB-Seq data across 2-Mb genomic bins in prostate. ρ represents the Spearman correlation coefficient. **c**, Scatter plots of 5hmC RPKM against CpG numbers across 2-Mb genomic bins in different tissues. R represents the Pearson correlation coefficient.

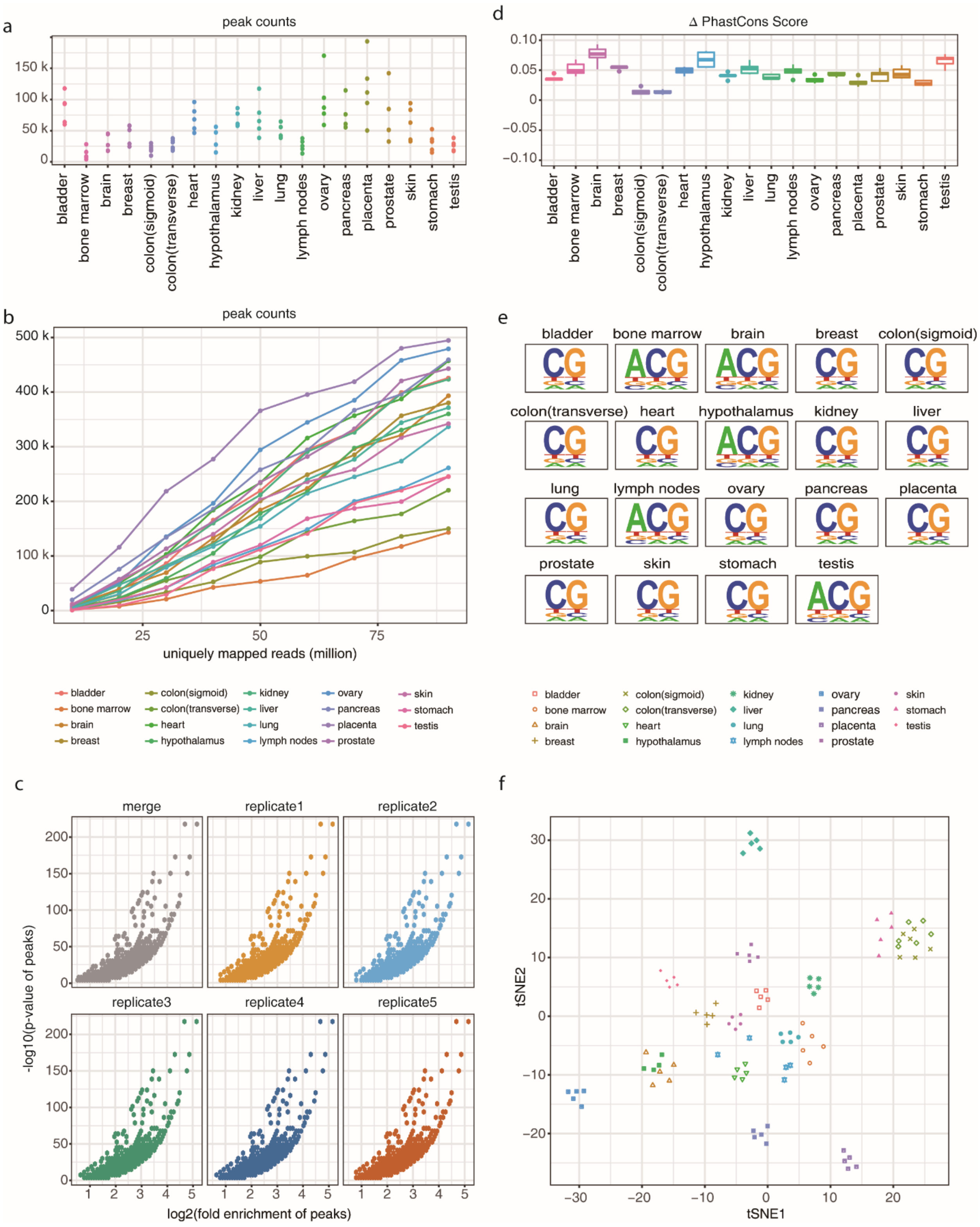
a
peak counts
150 k
100 k
50 k
0
bladder
bone marrow
brain
breast
colon(sigmoid)
colon(transverse)
heart
hypothalamus
kidney
liver
lung
lymph nodes
ovary
pancreas
placenta
prostate
skin
stomach
testis
d
Δ PhastCons Score
0.10
0.05
0
−0.05
−0.10
b
peak counts
500 k
400 k
300 k
200 k
100 k
0
25
50
75
uniquely mapped reads (million)
e
CG
ACG
c
merge
replicate1
replicate2
replicate3
replicate4
replicate5
200
150
100
50
0
1
2
3
4
5
-log10(p-value of peaks)
log2(fold enrichment of peaks)
f
30
20
10
0
−10
−20
−30
tSNE2
tSNE1

Supplementary Figure 2. Characteristics of 5hmC peaks. a, Numbers of 5hmC peaks across different tissues. Individual dots on the lines indicate different donor samples. **b**, Saturation curves showing 5hmC peak numbers against uniquely mapped reads for different tissues. **c**, Distributions of p-values and fold enrichments of 5hmC peaks in independent and merged prostate samples. **d**, Conservation status of 5hmC peaks minus control peaks based on PhastCons score. N= 5 biologically independent samples were used (n=4 for hypothalamus and n=6 for sigmoid and transverse colon). For all boxplots, center line represents median, bounds of box represent 25th and 75th percentiles and whiskers are Tukey whiskers. **e**, Top significant motifs under 5hmC peaks across different tissues. **f**, t-SNE clustering of genomic 5hmC distributions on 5hmC peaks for all donor tissue samples. Colored symbols indicate the organ/tissue associated with each 5hmC profile.

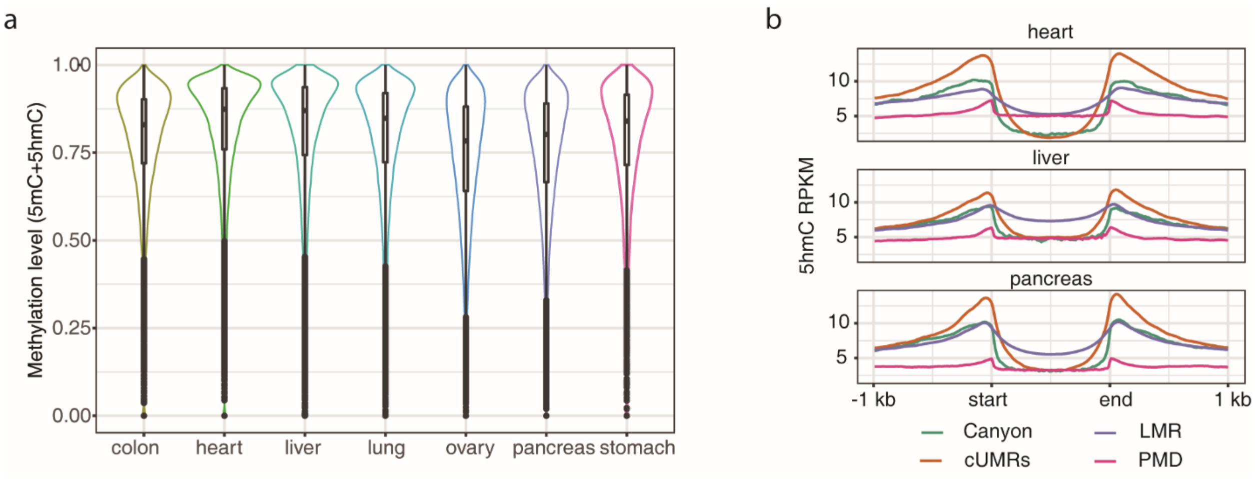

Supplementary Figure 3. Distribution patterns of 5hmC and 5mC. a, Methylation level (5mC + 5hmC) distributions across 5hmC peaks in 8 representative tissue types. N = 22,511 high-quality 5hmC peaks were used for colon, n = 35,828 for heart, n = 51,959 for liver, n = 30,063 for lung, n = 77,029 for ovary, n = 57,519 for pancreas, and n = 21,755 for stomach. For all boxplots, center line represents median, bounds of box represent 25th and 75th percentiles and whiskers are Tukey whiskers. **b**, 5hmC signals across different categories of the genome based on bisulfite sequencing. Canyons: DNA methylation canyons; cUMRs: control unmethylated regions; PMD: partially methylated domains; LMR: lowly methylated regions.

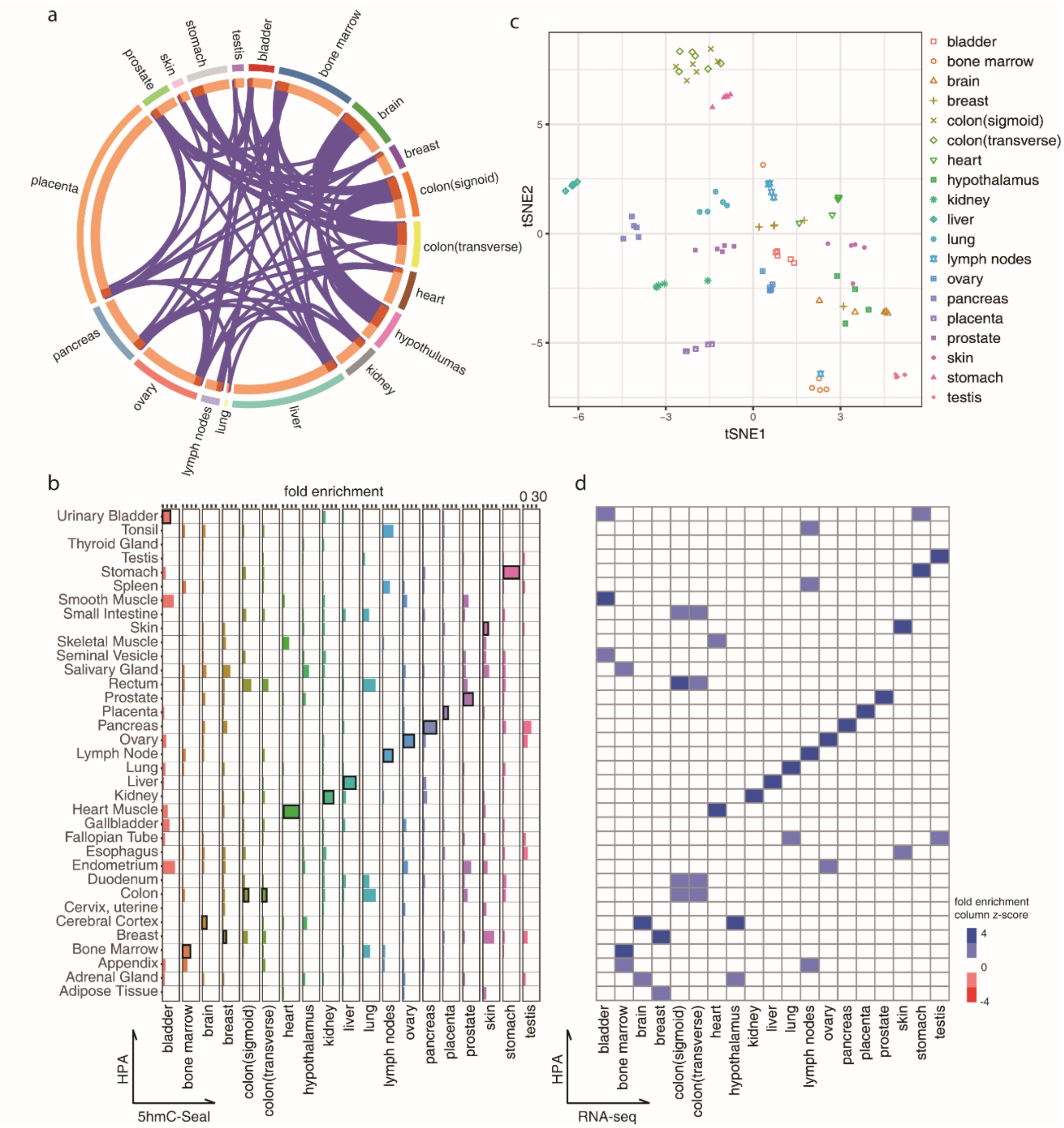

Supplementary Figure 4. Tissue-specific genes identified by 5hmC profiles and RNA-Seq.

a, Shared membership amongst different tissue-specific 5hmC modified genes. **b**, Fold enrichment of tissue-specific, 5hmC-modified genes with tissue-specific expressed genes from Human Protein Atlas. Bars with black boxes are concordant tissue types from Human Protein Atlas. **c**, t-SNE clustering of expression profiles on gene bodies for all donor tissue samples.

Colored symbols indicate the organ/tissue associated with each expression profile. **d**, Enrichment of our tissue-specific expressed genes via RNA-seq with those defined by Human Protein Atlas project.

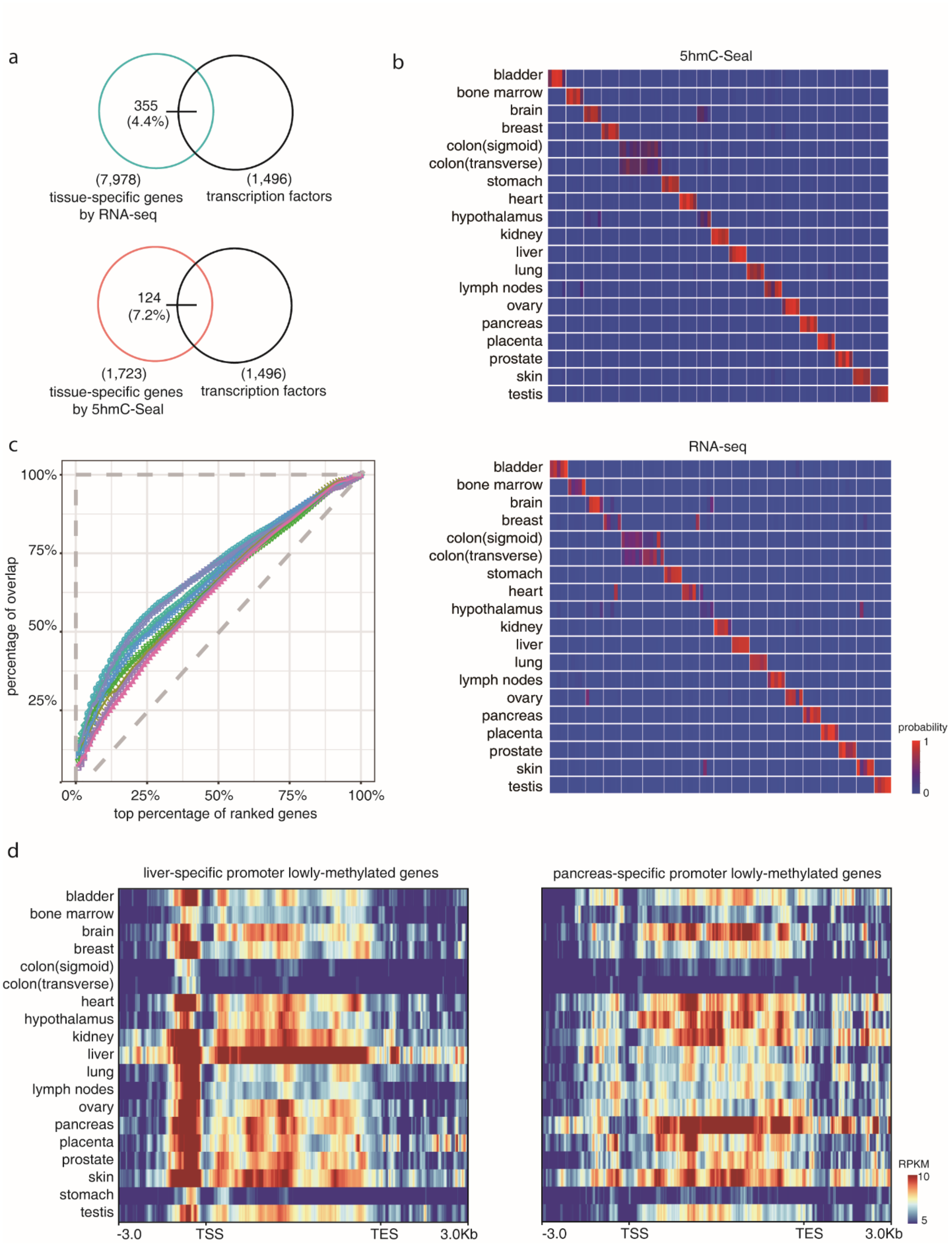
a
355
(4.4%)
(7,978)
tissue-specific genes
by RNA-seq
(1,496)
transcription factors
124
(7.2%)
(1,723)
tissue-specific genes
by 5hmC-Seal
(1,496)
transcription factors
b
5hmC-Seal
RNA-seq
bladder
bone marrow
brain
breast
colon(sigmoid)
colon(transverse)
stomach
heart
hypothalamus
kidney
liver
lung
lymph nodes
ovary
pancreas
placenta
prostate
skin
testis
probability
1
0
c
100%
75%
50%
25%
percentage of overlap
0%
25%
50%
75%
100%
top percentage of ranked genes
d
liver-specific promoter lowly-methylated genes
pancreas-specific promoter lowly-methylated genes
-3.0
TSS
TES
3.0Kb
RPKM
10
5

Supplementary Figure 5. Enrichment of 5hmC on tissue-specific genes. a, Overlaps between transcription factor genes and tissue-specific genes defined by 5hmC-Seal or RNA-seq. **b**, Multiclass regression models generated from 5hmC profiles and RNA-seq expression profiles for each of the 19 tissue types. The heatmap shown indicates different probability of model prediction. **c**, Correspondence at the top (CAT) plot showing percentages of gene overlap against top percentages of 5hmC-modified genes and H3K36me3-modified genes. **d**, Heatmaps showing tissue-specific, promoter lowly-methylated genes possessing the highest 5hmC signals in liver and pancreas tissues. Colors indicates RPKM values. TSS, transcription start site. TES, transcription end site.

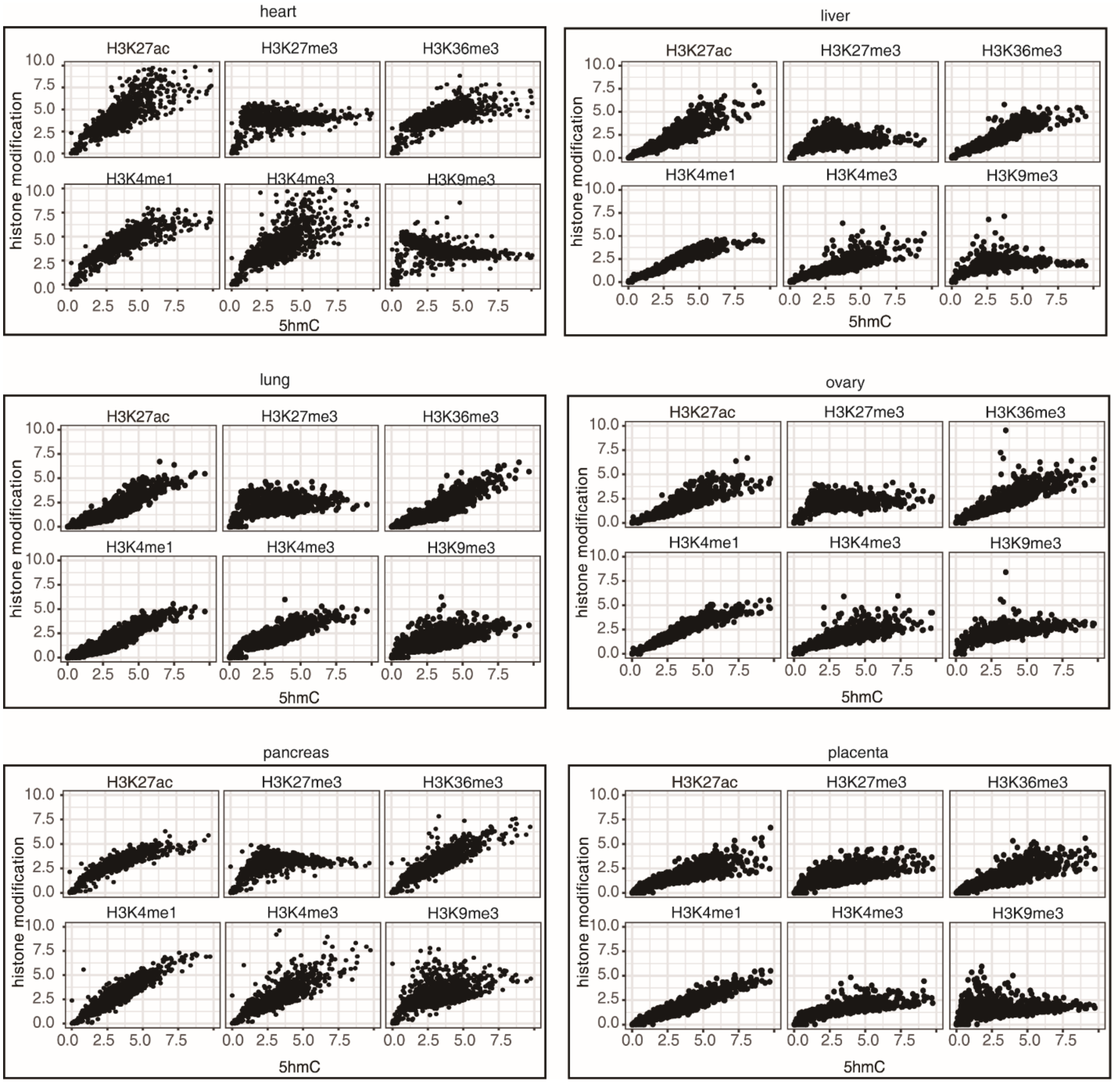

Supplementary Figure 6. Correlations of 5hmC profiles with other epigenomic data. Scatter plots for 2-Mb bins across the genome are shown.
