# Supplementary material for: A human tissue map of 5-hydroxymethylcytosines exhibits tissue specificity through gene and enhancer modulation
Source: Nat Commun. 2020 Dec 2;11:6161. doi: 10.1038/s41467-020-20001-w (PMC7710742; doi:10.1038/s41467-020-20001-w)
Supplement: Supplementary file 5 — Description of Additional Supplementary Files [file 41467_2020_20001_MOESM5_ESM.pdf]

Description of Additional Supplementary Files

Supplementary Data 1
Patient and 5hmC-Seal quality control information for all 96 samples.

Supplementary Data 2
Functional enrichment of tissue-specific 5hmCmodified genes.

Supplementary Data 3
Transcription factor genes covered by tissue-specific 5hmC-modified genes or tissue-specific expressed genes.
