# Supplementary material for: A human tissue map of 5-hydroxymethylcytosines exhibits tissue specificity through gene and enhancer modulation
Source: Nat Commun. 2020 Dec 2;11:6161. doi: 10.1038/s41467-020-20001-w (PMC7710742; doi:10.1038/s41467-020-20001-w)
Supplement: Supplementary file 6 — Reporting Summary [file 41467_2020_20001_MOESM6_ESM.pdf]

nature research

Corresponding author(s): Chuan He

Last updated by author(s): Oct 31, 2020

Reporting Summary

Nature Research wishes to improve the reproducibility of the work that we publish. This form provides structure for consistency and transparency in reporting. For further information on Nature Research policies, see Authors & Referees and the Editorial Policy Checklist .

Statistics

For all statistical analyses, confirm that the following items are present in the figure legend, table legend, main text, or Methods section.

n/a	Confirmed	
☐	☒	The exact sample size (*n*) for each experimental group/condition, given as a discrete number and unit of measurement
☐	☒	A statement on whether measurements were taken from distinct samples or whether the same sample was measured repeatedly
☐	☒	The statistical test(s) used AND whether they are one- or two-sided<br>*Only common tests should be described solely by name; describe more complex techniques in the Methods section.*
☒	☐	A description of all covariates tested
☐	☒	A description of any assumptions or corrections, such as tests of normality and adjustment for multiple comparisons
☐	☒	A full description of the statistical parameters including central tendency (e.g. means) or other basic estimates (e.g. regression coefficient) AND variation (e.g. standard deviation) or associated estimates of uncertainty (e.g. confidence intervals)
☐	☒	For null hypothesis testing, the test statistic (e.g. *F*, *t*, *r*) with confidence intervals, effect sizes, degrees of freedom and *P* value noted<br>*Give P values as exact values whenever suitable.*
☒	☐	For Bayesian analysis, information on the choice of priors and Markov chain Monte Carlo settings
☒	☐	For hierarchical and complex designs, identification of the appropriate level for tests and full reporting of outcomes
☐	☒	Estimates of effect sizes (e.g. Cohen's *d*, Pearson's *r*), indicating how they were calculated

Our web collection on statistics for biologists contains articles on many of the points above.

Software and code

Policy information about availability of computer code

Data collection	No software was used.
Data analysis	Trim Galore (v0.4.4); Bowtie (v1.2.3); samtools (v1.9); bedtools (v2.28.0); deeptools (v3.3.1); featureCounts (v1.6.2);HISAT2 (v2.1.0); MACS2 (v2.2.5); R (v3.6.1); MethylSeekR (v1.28.0); HPA algorithm (v19)

For manuscripts utilizing custom algorithms or software that are central to the research but not yet described in published literature, software must be made available to editors/reviewers. We strongly encourage code deposition in a community repository (e.g. GitHub). See the Nature Research guidelines for submitting code & software for further information.

Data

Policy information about availability of data

All manuscripts must include a data availability statement. This statement should provide the following information, where applicable:

- Accession codes, unique identifiers, or web links for publicly available datasets
- A list of figures that have associated raw data
- A description of any restrictions on data availability

The raw and processed 5hmC-Seal and RNA-seq data have been deposited into the NCBI Gene Expression Omnibus (GEO) and are accessible through GEO series accession number GSE144530 (https://www.ncbi.nlm.nih.gov/geo/query/acc.cgi?acc=GSE144530). Public TAB-seq dataset used in this study was downloaded from GSE104780 (https://www.ncbi.nlm.nih.gov/geo/query/acc.cgi?acc=GSE104780). Genomic Segments dataset based on WGBS data was downloaded from GSE113405 (https://www.ncbi.nlm.nih.gov/geo/query/acc.cgi?acc=GSE113405). Chromatin states and enhancers data were directly downloaded from Roadmap Epigenomics Project website (http://www.roadmapepigenomics.org).

Field-specific reporting

Please select the one below that is the best fit for your research. If you are not sure, read the appropriate sections before making your selection.

☒ Life sciences ☐ Behavioural & social sciences ☐ Ecological, evolutionary & environmental sciences

For a reference copy of the document with all sections, see nature.com/documents/nr-reporting-summary-flat.pdf

Life sciences study design

All studies must disclose on these points even when the disclosure is negative.

Sample size	No statistical methods were used to predetermine sample size. For sequencing data, sample size were determined based on our prior experience on similar experiments and literature reports (Stark R., Hadfield J. (2016)). Basically, three or four replicates of each sample type for in vitro experiments are needed, and more biological replicates are required for in vivo experiments such as primary tissues from patients. We performed all experiments with four to six samples to make sure results are consistent.
Data exclusions	The information about blacklisted genomic regions for functional genomics analysis were provided by the ENCODE project.
Replication	Results were confirmed in four to six biological replicates for tissue types. All attempts to replicate data are successful.
Randomization	All samples were randomly chosen to sequence in six batches.
Blinding	The investigators were blinded during quality assessment of raw sequencing data.

Reporting for specific materials, systems and methods

We require information from authors about some types of materials, experimental systems and methods used in many studies. Here, indicate whether each material, system or method listed is relevant to your study. If you are not sure if a list item applies to your research, read the appropriate section before selecting a response.

Materials & experimental systems

n/a	Involved in the study	
☒	☐	Antibodies
☒	☐	Eukaryotic cell lines
☒	☐	Palaeontology
☒	☐	Animals and other organisms
☐	☒	Human research participants
☒	☐	Clinical data

Methods

n/a	Involved in the study	
☒	☐	ChIP-seq
☒	☐	Flow cytometry
☒	☐	MRI-based neuroimaging

Human research participants

Policy information about studies involving human research participants

Population characteristics	All the tissue donors were adults of European ancestry, and 48% were males, with an average age of 52 years.
Recruitment	Death of individuals was generally due to accident or cause unrelated to the tissue collected and post-mortem interval was <6 hours.
Ethics oversight	Either donors or next of kin were consented for DNA, RNA and protein analysis through an ethics board approved study protocol at Proteogenex.

Note that full information on the approval of the study protocol must also be provided in the manuscript.
